# Factors associated with the disbursements of development assistance for health in low-income and middle-income countries, 2002–2017

**DOI:** 10.1136/bmjgh-2020-004858

**Published:** 2021-04-23

**Authors:** Modhurima Moitra, Ian Cogswell, Emilie Maddison, Kyle Simpson, Hayley Stutzman, Golsum Tsakalos, Joseph Dieleman, Angela E Micah

**Affiliations:** 1 Department of Health Metrics Sciences, University of Washington School of Medicine, Seattle, Washington, USA; 2 Institute for Health Metrics and Evaluation, University of Washington, Seattle, Washington, USA

**Keywords:** health economics

## Abstract

**Introduction:**

In 2017, development assistance for health (DAH) comprised 5.3% of total health spending in low-income countries. Despite the key role DAH plays in global health-spending, little is known about the characteristics of assistance that may be associated with committed assistance that is actually disbursed. In this analysis, we examine associations between these characteristics and disbursement of committed assistance.

**Methods:**

We extracted data from the Creditor Reporting System of the Organization for Economic Co-operation and Development, Institute for Health Metrics and Evaluation, and the WHO National Health Accounts database. Factors examined were off-budget assistance, administrative assistance, publicly sourced assistance and assistance to health systems strengthening. Recipient-country characteristics examined were perceived level of corruption, civil fragility and gross domestic product per capita (GDPpc). We used linear regression methods for panel of data to assess the proportion of committed aid that was disbursed for a given country-year, for each data source.

**Results:**

Factors that were associated with a higher disbursement rates include off-budget aid (p<0.001), lower administrative expenses (p<0.01), lower perceived corruption in recipient country (p<0.001), lower fragility in recipient country (p<0.05) and higher GDPpc (p<0.05).

**Conclusion:**

Substantial gaps remain between commitments and disbursements. Characteristics of assistance (administrative, publicly sourced) and indicators of government transparency and fragility are also important drivers associated with disbursement of DAH. There remains a continued need for better aid flow reporting standards and clarity around aid types for better measurement of DAH.

**Key questions**

**What is already known?**

- Development assistance for health (DAH) is an important source of health funding for low-and-middle-income countries.
- There are existing discrepancies in the proportion of committed DAH that gets disbursed.
- Different data sources use different metrics to measure DAH which poses challenges to accurately examining disbursement trends.

**What are the new findings?**

- DAH resources that have greater than 15% attributed to administrative expenses is associated with lower disbursed assistance relative to commitment.
- DAH resources that have greater than 25% attributed as off-budget delivery is associated with a higher disbursed assistance relative to commitment.
- Greater public trust and lower perception of corruption is associated with higher disbursed assistance relative to commitment.
- Higher political fragility is associated with lower disbursed assistance relative to commitment.
- There is variation in disbursed assistance relative to commitment by geographical region and income group of recipient countries.

**What do the new findings imply?**

- Characteristics of assistance such as the administrative expense associated with delivery of aid and the governance indicators of perceived corruption or fragility are key indicators of the likelihood of disbursements relative to committed assistance.
- There is a continued need for more clarity around flows of assistance and standardised reporting mechanisms for DAH.

## Introduction

In 2019, development assistance for health (DAH) made up 3.9% of total health spending in lower-middle-income countries and 5.3% of total health spending in low-income countries.^1^ DAH, defined as the financial and non-financial resources that are transferred through international development agencies with the primary intent of promoting and maintaining health in low-and-middle-income countries, thereby comprises an essential source of funding for health for specific vulnerable countries globally. More recently, advocates for development assistance contributions have suggested that another critical role for those funds to play is towards the provision of global public goods such as health systems strengthening, surveillance systems, research and development for neglected tropical diseases.^2 3^ These are goods for which there are long-term transnational benefits but may be difficult to hold individual countries accountable for its provision.

Despite the key role DAH plays in health spending in low-resource settings, the effectiveness and clarity of its allocation methods have been called into question. Critiques argue that the allocation strategy used by major international development agencies to distribute aid are not aligned to key criteria such as disease burden or humanitarian need but are more aligned to country national income which may mask wide within country wealth disparities, donor’s own priorities, commercial or political interests, and historic ties.^3–15^ Aid flows are also reported in different ways that limit our understanding of a comprehensive summary of all sources of aid.^3 16–19^ Studies often use varying metrics to define aid (either commitments or disbursements or expenditure) which may provide differing estimates of aid and represent different stages of the administration of aid. Despite joint agreements made on principles of more effective and equitable aid delivery through the 2005 Paris Declaration and the 2008 Accra Agenda for Action, disbursement of development assistance could be made less fragmented with more coordination and improved accountability or transparency in operations^4 5^ More broadly, the effectiveness of aid has also been called into question on its ability to move populations out of poverty.^3 20 21^ The large body of existing evidence suggests that the pathway for translating aid into positive outcomes is complex and may be influenced by such factors as the quality of institutions, availability of human capital, infrastructure and the economic and policy environment in recipient countries and the quality of aid from the donor perspective although it ultimately remains inconclusive.^22–25^

There is limited although insightful literature examining patterns of development assistance and the extent to which aid commitments are realised in actual disbursements or expenditures. McCoy *et al* examined major sources, management and spending mechanisms of development assistance and found that while development assistance has increased in recent years, it is also accompanied by lack of coordination, accountability and adequate monitoring of global health finances.^17^ This poses challenges for governments of low-and-middle-income countries (LMICs) and makes it difficult to obtain clarity around management and dissemination of global health funds. An analysis of aid commitments and disbursements to multiple sectors including health from 2002 through 2010 found that although on average most commitments are disbursed within 2 years, there are differences in disbursement patterns for individual sectors with some sectors having very long lags.^18^ An analysis of official development assistance for health showed significant imbalances in the allocation of aid with inadequate funding for many health priorities (other than HIV/AIDs, malaria, other diseases).^3 26–28^ Van de Maele *et al* examined aid flows for sub-Saharan African countries from 2002 to 2011 by making use of three data sources, namely the Organization for Economic Cooperation and Development’s Creditor Reporting System (OECD), the Institute for Health Metrics and Evaluation’s development assistance for health data (IHME DAH), and WHO’s National Health Accounts (NHA).^16^ Their analyses showed discrepancies in commitments and disbursements by data source and variation at the donor or recipient country level.^16^

Based on the findings in previous studies described earlier, it is important to examine factors that may contribute to variation in disbursement patterns over time and location. For instance, the proportion of aid that is made in-kind or as off-budget may impact proportion of committed aid that gets disbursed. Odedokun presents an analysis focused on the determinants of deviations in disbursements and delays in development assistance allocations from the donor perspective. The study findings show that a higher number of checks and balances in the political system is one of the factors associated with a lower fraction of aid disbursed. Also, donors who give more aid in the form of grants disburse less of their commitments compared with other donors.^29^ From the recipient country perspective, recipient country-level factors such as public perception of corruption and risk of political fragility may also be associated with variation in disbursements.^30–32^ While these factors have been studied previously, there has been limited analysis that include administrative expenses. Easterly and Pfutze highlight overhead costs as one of factor that could be improved in aid delivery.^33^ In this analysis, we examine associations between these potential drivers and the proportion of disbursed aid.

## Methods

We used three main sources of data for the analyses: The Organization for Economic Cooperation and Development’s Creditor Reporting System (CRS), the Institute for Health Metrics and Evaluation’s Financing Global Health Development Assistance for Health (FGH DAH) and WHO’s National Health Accounts (NHA) data reported in the Global Health Expenditure Database. We used data from all three sources because each captures a different stage of the resource flow pathway. From the CRS, we extracted gross commitment and gross disbursement data for all 30 countries on the Development Assistance Committee. We limited projects to those in the general health, basic health, and population and reproductive health programme sectors from the OECD CRS online database. Since data coverage and quality have increased over time, we used data from 2002 to 2017 to maximise data availability and consistency with other data sources. From the IHME DAH database, we pulled DAH estimates for disbursements and commitments that were disaggregated into programmatic and administrative expenses. From the GHED National Health Accounts Data (NHA), two indicators—(1) rest of the world (RoW) expenditures which is defined as the amount of health expenditure that is through non-resident financing schemes and (2) transfers distributed by governments that are of foreign origin—were extracted. We estimated the disbursement rate relative to commitment amount as the ratio of disbursement to commitment for each of the aforementioned data sources. As such, for a given country-year, ratios closer to 0 indicated low disbursement to commitment whereas ratios closer to 1 indicated higher disbursement to commitment levels. The independent variables used in the analyses are described in table 1.

**Table 1:** List of variables and definitions

Independent variable	Definition
Off-budget delivery >25%*	Indicator for proportion of aid that is delivered outside government systems that are greater than 25%
Corruption Perception Index (CPI)†	CPI scores measure of the perceived level of corruption in a country’s public sector. Values range from 0 (most corrupt) to 100 (least corrupt)^41^
Fragile States Index (FSI)‡	A measure of the risk of conflict and vulnerabilities faced by individual nations. Values range from 0 (least fragile) to 120 (most fragile)^42^
Proportion of public DAH	The proportion of DAH in a country-year that is from public sources
In-Kind DAH >15%*	Indicator for DAH administrative expenses values that are greater than 15%
Gross domestic product	Logged GDP per capita for each country-year (GDPpc)
Proportion of DAH allocated to health systems strengthening	The proportion of DAH in a country-year that is allocated to health systems strengthening projects (DAH_HSS)
Proportion of DAH from non-governmental sources	The proportion of DAH in a country-year that is from non-governmental sources
GBD super-region (reference: Central Europe, Eastern Europe and Central Asia)	Countries were classified as belonging to one of 7 super regions as per the Global Burden of Disease Study^15^
Income level	Countries were classified as belonging to one of the four income levels as designated by the World Bank^43^

*The threshold for off-budget delivery were chosen to be 25% as most NHA expenditure data had between 0% and 25% allocated as off-budget. Similarly, the threshold for administrative expenses (in-kind) was chosen to be 15% as most DAH in this dataset had between 0% and 15% allocated as administrative expenses.

†CPI scores are published by Transparency International.

‡FSI composite scores used here are published by the Fund For Peace.

A panel linear regression model with country fixed-effects was used to examine the relationship between the independent variables of interest and the outcome variable. This empirical approach suited the collated dataset because it comprised repeated measurements over time. A log-transformation of our outcome variable was initially tested, but while it did not change the main results, it made the interpretation of results less accessible. Therefore, we modelled our outcome variable on its original scale. Since sampling error is correlated with time, we included a random-effects term for our time variable (year) and fixed effects for our remaining covariates. Sensitivity analyses were conducted to test for cross-sectional dependence using the Breusch-Pagan Lagrange multiplier test of independence to check for residual correlation across panels. Heteroscedasticity across panels was controlled for using Huber-White sandwich estimators for robust SEs and for fixed effects using heteroscedasticity and autocorrelation-consistent (HAC) SEs. The formally notated equation is provided later.

We examined the associations using data from three sources as previously indicated. First, the OECD Creditor Reporting System provided data on both commitments and disbursements, which we used to calculate the proportion of CRS-reported disbursement to commitment. Second, we checked the robustness of the results by using disbursement and commitment data from the IHME database to calculate the proportion of IHME-estimated disbursement to commitment. As an additional source of comparison, we also used the expenditure data from the national health accounts to calculate the proportion of NHA expenditure to CRS commitment. Additional relevant variables of interest were analysed to check for significant associations. The three data sources used provide a snapshot of DAH flows at different stages.

$$y_{it}=\alpha +\beta^{\prime }X_{it}+e_{it}+u_{i}$$

where y_i_ denotes ratios i=1… N and t=1… T, $\beta^{\prime }$ is the vector of coefficients for chosen predictors, e_it_ denotes the sampling error and u_i_ represents year-level random effects

## Results

**Figure 1:**
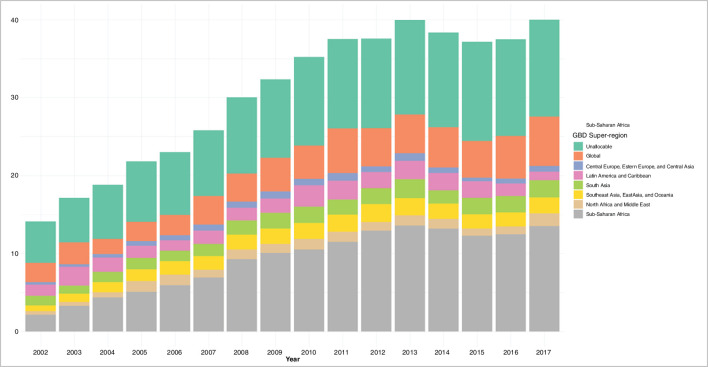
IHME development assistance for health (DAH) by year and GBD super-region. Note: Development assistance for health in US dollars (billions) disaggregated by GBD super-regions for years 2002–2017. Additional categories include DAH that is disbursed at the global level and DAH that is unallocable. GBD super-regions are regions that group sub-regions based on cause of death patterns. These are GBD high-income, Latin America and the Carribbean, sub-Saharan Africa, Southeast Asia, East Asia and Oceania, South Asia and North Africa and the Middle East.

**Figure 2:**
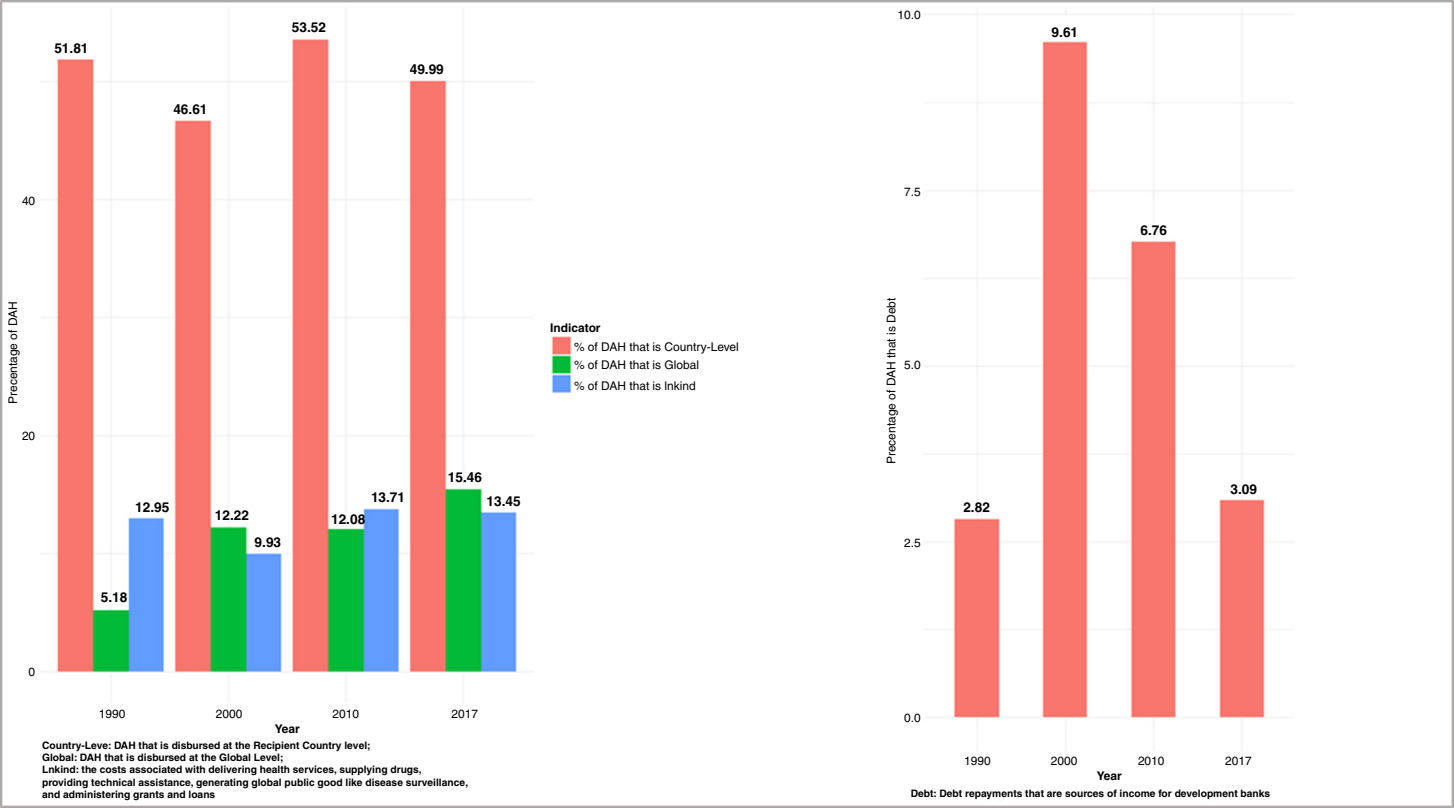
Proportion of IHME development assistance for health (DAH) allocated to select components. Left panel: proportion of DAH that is disbursed at the recipient country level, global level and as in-kind administrative expenses. Right panel: proportion of DAH that is disbursed as debt repayments that are a source of income for development banks.

**Figure 3:**
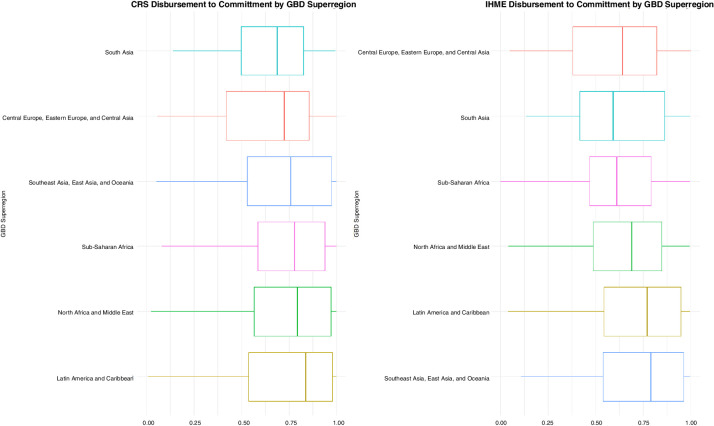
Proportion of development assistance for health (DAH) disbursement to commitment by GBD super-region. Note: Proportion of DAH disbursement to commitment disaggregated by GBD super-regions.

Figure 1 highlights the growth in DAH from 2002 through 2017 allocated to six GBD super-regions from member countries of the Development Assistance Committee (DAC). From 2002 through 2017, a total of US$106.3 billion has been contributed towards activities that maintain or improve health in LMICs in these regions. In 2017, the latest year of analysis in this study, DAH amounted to US$40 billion, which is a nearly threefold increase from its value in 2002. The share of DAH allocated to country-level projects, administrative expenses, global initiatives and from debt repayments over time are shown in figure 2. The majority of DAH was allocated to country-level projects with lesser proportions allocated to global, administrative expenses, respectively. While the percentage for country-level and administrative expenses have remained roughly the same over time, the percentage targeted towards global activities has at least doubled over time. The proportion of committed DAH that was disbursed in the IHME and CRS data are shown in figure 3. Median disbursement varied by source. Latin America and the Caribbean had the highest median disbursement per the CRS dataset while in the IHME dataset, Southeast Asia was the region with the highest median disbursement. We present regression results using ratios from 0 to 1 here modelled as a function of CPI, administrative expenses greater than 15%, off-budget delivery greater than 25%, GDP per person, development assistance targeted towards health system strengthening, development assistance channelled through non-governmental sources, global burden of disease super-region and income level with random effects for year. Regression coefficients and SEs are reported in table 2. Results using the Fragile States Index (FSI) as another exploratory indicator and income level are reported in online supplemental appendix.

10.1136/bmjgh-2020-004858.supp1 Supplementary data

**Table 2:** Estimated coefficients and SEs from multiple regression models on the determinants of the proportion of committed development assistance for health that is disbursed

Parameter	CRS	IHME	NHA
Intercept	0.66 (0.03)***	0.64 (0.05)***	0.34 (0.04)***
Off-budget >25%	–	–	0.12 (0.06)*
In-kind >15%	–	−0.06 (0.02)***	–
CPI	0.002 (0.001)*	0.003 (0.001)***	0.003 (0.001)*
Per cent HSS DAH	–	−0.031 (0.03)	–
Per cent NGO DAH	–	−0.03 (0.02)	–
GDP per capita	0.015 (0.01)	0.02 (0.01)*	−0.04 (0.014)**
Per cent DAH from public sources	–	0.01 (0.04)	–
Latin America and Caribbean	0.06 (0.03)	0.07 (0.03)	−0.003 (0.04)
North Africa and Middle East	0.09 (0.04)*	0.05 (0.03)	0.09 (0.05)
South Asia	−0.005 (0.05)	0.001 (0.04)	0.21 (0.17)
Southeast Asia, East Asia and Oceania	0.05 (0.03)	0.11 (0.03)***	0.09 (0.05)
Sub-Saharan Africa	0.085 (0.03)**	0.04 (0.02)	0.097 (0.04)*

CRS (n=1034); IHME (n=1119); NHA (n=1089).

GBD super-region reference level: Central Asia, Central Europe, Eastern Europe.

*Significance codes: 0 ‘***’ 0.001 ‘**’ 0.01 ‘*’ 0.05.

The results based on CRS data by GBD super-region show a significant association between an increase in the Corruption Perception Index (CPI) scores and the proportion of DAH disbursed. The proportion of disbursements increased by 0.02 units for each 10-unit increase in CPI score. DAH to Southeast Asia along with North Africa and Sub-Saharan Africa are also associated with significantly higher disbursement compared with the reference group (Central Asia, Central and Eastern Europe). Relative to the reference group, a 10-point unit increase is associated with the proportion of disbursements increasing by 0.9 units for disbursements in North Africa and Middle East and in Sub-Saharan Africa, respectively. The regression results using IHME DAH data show that increase in CPI scores and GDP were associated with an increase in DAH disbursed, whereas DAH disbursed with administrative expenses greater than 15% was associated with lower DAH disbursement. DAH to Southeast Asia was also associated with an increase in the proportion of DAH disbursed.

In the results using NHA expenditure data, off-budget expenditure greater than 25% and an increase in CPI scores were associated with an increase in the proportion of DAH disbursed. An increase in off-budget expenditure greater than 25% was associated with an increase of 0.12 units in disbursement of committed assistance. DAH to sub-Saharan Africa was also associated with an increase in proportion of DAH disbursed.

Additional results reported in online supplemental appendix show that FSI scores were also significantly associated with IHME DAH disbursement indicating that greater fragility was associated with lower disbursement. In results by income level, DAH allocated to LMICs was associated with the highest relative increase in DAH disbursed compared with upper-middle-income recipients (see online supplemental appendix).

## Discussion

This study used data from three sources—OECD CRS, IHME DAH and NHA expenditure data—to examine the factors that are associated with the likelihood of aid commitments being disbursed. The key findings highlight that while development assistance for health grew significantly in the first decade of the 21st century, it has since plateaued. Furthermore, while a portion of development assistance for health goes towards operational expenses related to loan administration and about a quarter may be delivered outside government systems, a reasonable percentage of aid goes towards programmatic activities. Most importantly, the results show that administrative expenses associated with aid delivery, off-budget delivery, CPI and regional/income-level variation are associated with the likelihood of aid commitments being disbursed.

Historically, development assistance for health has been tracked from the donor perspective so while there is a general awareness of the sources of assistance, the availability of such information from the recipient country perspective is limited.^34^ This is a critical gap that needs to be filled because previous tracking efforts as well as results from this study have shown that there is often a difference between what is initially committed, what might be disbursed and what is ultimately spent in country.^16^ While these differences may not be inherently problematic, it is useful to understand where there might be opportunities to improve on the efficiency of transfer of resources across entities.

Furthermore, the results reported here demonstrate that select factors may be associated with variation in disbursement patterns. Although patterns of disbursement vary over time, substantial gaps between commitment and disbursement remain at the regional and income group levels. For example, we see that administrative expenses (above 15% of total DAH) are associated with lower disbursement of DAH. Administrative expenses as defined for this study captures the operational expenses disbursing agencies incur while delivering the loan or grant. This suggests that project grants for which there are significant operational challenges also have challenges related to financial disbursements or use of the available funds. Bureaucratic policies both within the recipient country and donor country may limit the efficient flow of funds to support project activities. While there are very limited publicly available data on the operational expenses for aid agencies especially in regards to personnel, the challenges it presents for the efficient delivery of aid have been noted in the literature.^34^ Furthermore, many countries may apply certain conditions on types of aid received or proportion of domestic co-financing and may delay disbursement if those conditions are not met.^14^ Administrative expenses tied to DAH may pose a burden to the capacities of countries to use aid in a timely manner.^4^ Although many regions have enacted standardised procedures to increase efficiency and accountability in administrative processes, there remain gaps between principles and practice.^35^ Alternatively, countries for which there was less of a perception of corruption across time (increase in CPI scores) were associated with higher DAH disbursement levels of their committed DAH allocation for projects. CPI scores are indicative of public trust and legitimacy. Therefore, a positive association between CPI and DAH disbursement may be reflective of robust and transparent governmental procedures in recipient countries that enable and support ease of DAH disbursement. Increase in FSI scores (indicating greater fragility) on the other hand were associated with lower IHME DAH disbursement (see online supplemental appendix). This may reflect greater volatility in politically fragile countries that may impact effective disbursement of aid. These findings are consistent with other studies that find that greater transparency and scope for public deliberation are associated with improved consistency of funding.^14^ Higher GDP per capita was associated with an increase in disbursed DAH. It is plausible that higher GDP may be associated with greater stability and stronger governance and therefore better checks on money being disbursed for DAH.

Another factor that was associated with increased disbursement to commitment was instances in which majority of aid delivery (off-budget delivery greater than 25%) was channelled outside government systems. Off-budget support is typically delivered outside of government budgetary processes and therefore may be faster to disburse because they avoid government bureaucratic systems. However, the proportion of DAH from public sources was associated with lower DAH disbursement because these are also part of the government bureaucratic systems and may have more procedural requirements before resources can be released.^36^ This finding aligns with the earlier finding regarding limited disbursement due to high administrative expenses as discussed previously.

This study focused on factors associated with DAH disbursement from the recipient country as well as the donor country perspective. It is important to consider the effectiveness of delivery systems from both the donor and recipient perspective.^37^ While national income per person is a commonly used criterion by funders, it may not always align with existing health needs or inequities in the recipient country.^14^ In addition, quality of DAH in terms of its loan and grant composition may also be important to explore.^38 39^ Although this study does not directly measure the impact of quality, some covariates used in our analyses may be indicative of DAH quality. Lower administrative costs as well as higher allocations of assistance to well-governed countries and sharing aid with partners that have good operational strategies are indicators of high-quality development assistance in terms of maximising efficiency.^40^

The findings from this study highlight some areas of the aid delivery structure that may be addressed to increase the likelihood that more of the committed amounts for aid activities are disbursed to support planned activities. Specifically, as previous high-level meetings to deliberate aid effectiveness have highlighted, it is essential that donor and recipient countries continue to work on aligning, harmonising systems and minimising the bureaucracy associated with aid delivery. In addition, the finding that less corrupt systems have better disbursement ratios suggest that donors and recipients alike can proactively work towards limiting and address the gaps in the aid delivery systems that may present opportunities to misdirect funds to alternative use.

Some limitations are important to note for this study. All three data sources had different forms of aid data available. While these were valuable for examining the efficiencies along various stages of aid flow, it limited the types of additional factors that we explored across the models. Second, we were only able to use commitment data from the CRS for models using NHA data with the assumption that these commitments were the relevant ones. Lastly, in this analysis we explored associations so none of the associations can be interpreted as causal ones.

## Conclusion

Differences between DAH disbursement and commitments to LMICs continue to exist. These may be associated with both recipient country and donor-level factors as well as components of aid that are delivered. Robust monitoring systems improve reporting and tracking efforts, and also directly contribute to the effectiveness of aid.^41 42^ Therefore, there remains a need for better aid flow reporting standards and clarity around aid types for better measurement of DAH at both the donor and recipient levels.

## Data Availability

Data are available in a public, open access repository: IHME Development Assistance for Health database (http://ghdx.healthdata.org/record/ihme-data/development-assistance-health-database-1990-2019OECD); Creditor Reporting System (https://stats.oecd.org/Index.aspx?DataSetCode=crs1); WHO Global Health Expenditure Database (https://apps.who.int/nha/database/Select/Indicators/en).
