# Supplementary material for: Factors associated with the disbursements of development assistance for health in low-income and middle-income countries, 2002–2017
Source: BMJ Glob Health. 2021 Apr 23;6(4):e004858. doi: 10.1136/bmjgh-2020-004858 (PMC8074566; doi:10.1136/bmjgh-2020-004858)
Supplement: Supplementary data [file bmjgh-2020-004858supp001.pdf]

Appendix

Factors associated with development assistance for health disbursements in low- and middle-income countries, 2002-2017

Summary Distribution of Variables:

CPI: Corruption Perception Index (CPI) scores ranged from 0 through 100 with lower scores reflecting greater corruption higher scores reflecting lower corruption. CPI scores are summarized here by GBD super-region with lower median CPI scores for Southeast Asia and followed by North Africa compared to other super-regions.

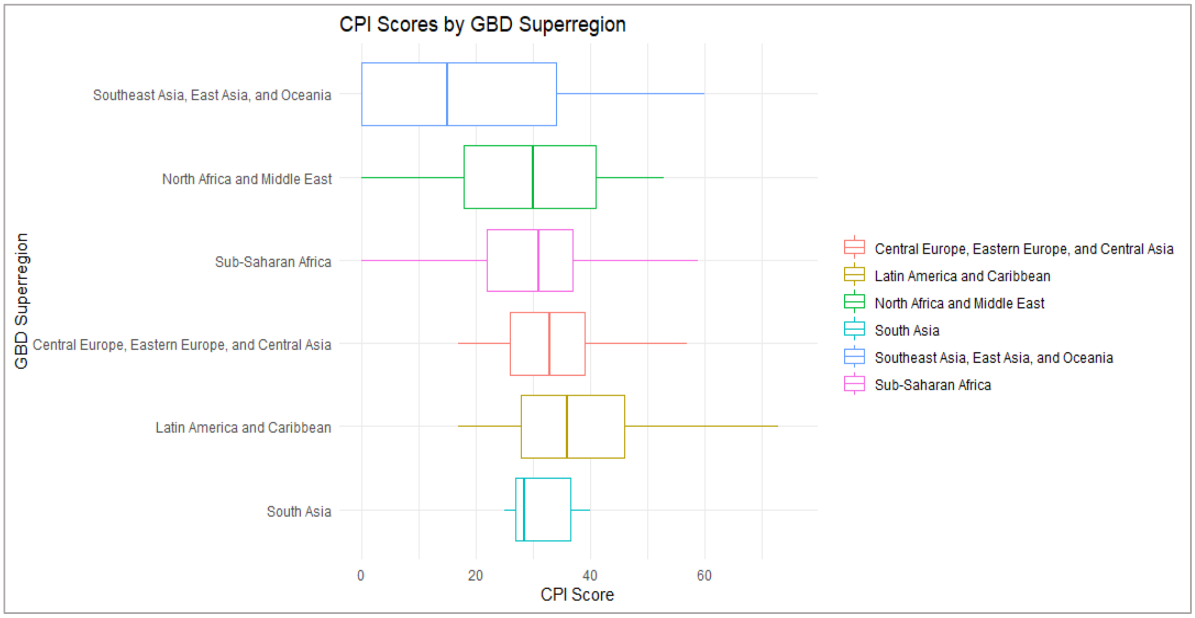

Moitra M, *et al. BMJ Global Health* 2021; 6:e004858. doi: 10.1136/bmjgh-2020-004858

FSI: Fragile States Index scores ranged from 0 through 120 with higher scores reflecting higher fragility. FSI scores summarized here show higher relative median FSI scores for South Asia, North Africa, Sub-Saharan Africa, followed by the remaining super-regions.

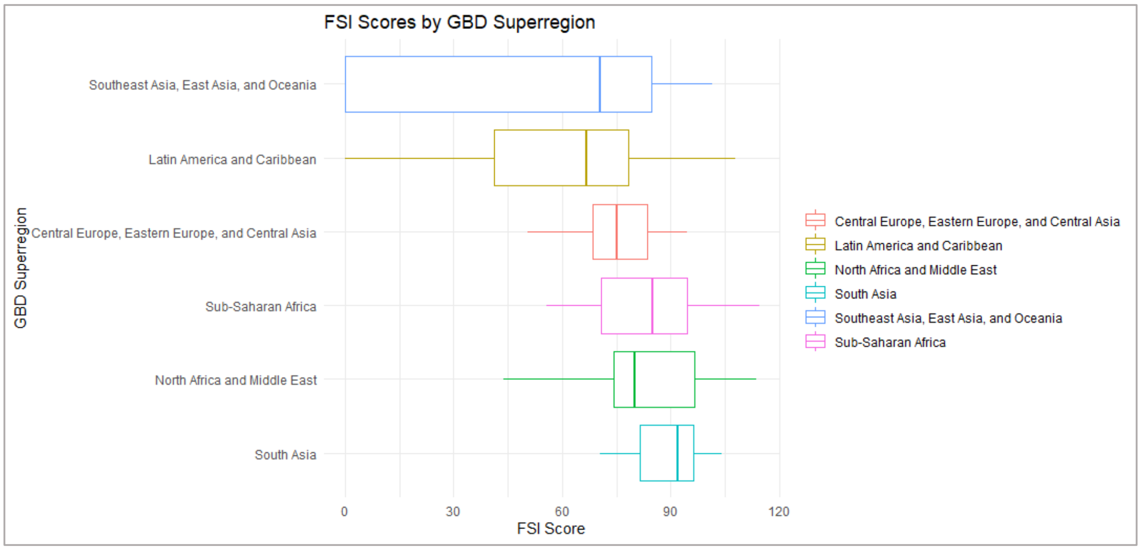

In-Kind > 15%: In-Kind or administrative expenses were calculated as a proportion of total DAH. Median proportions of In-Kind DAH were similar for most GBD super-regions – between 10 to 15 percent.

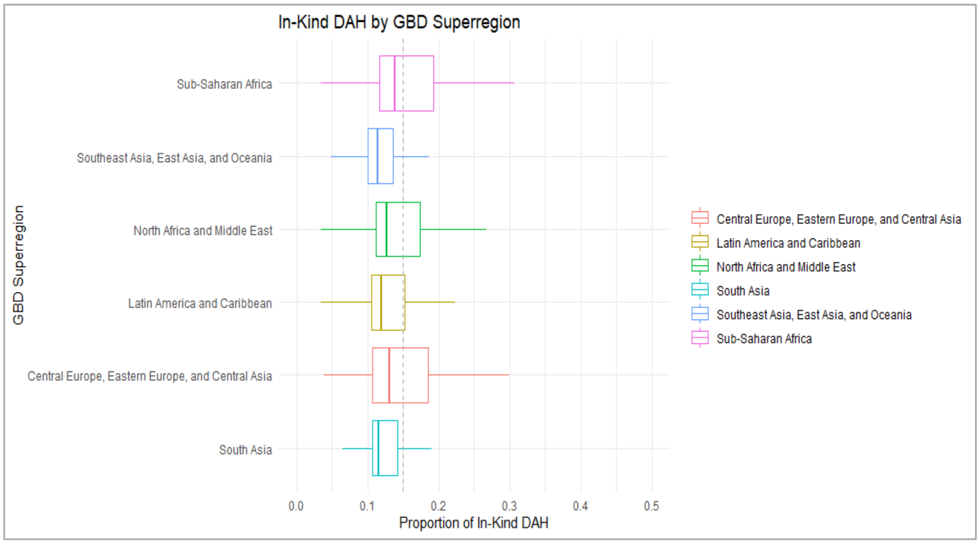

Off-budget > 25%: Off-budget delivery as a proportion of total expenditure is summarized here by GBD super-region. Off-budget delivery was characteristic of NHA expenditure predominantly for Southeast Asia, South Asia, and Central Europe.

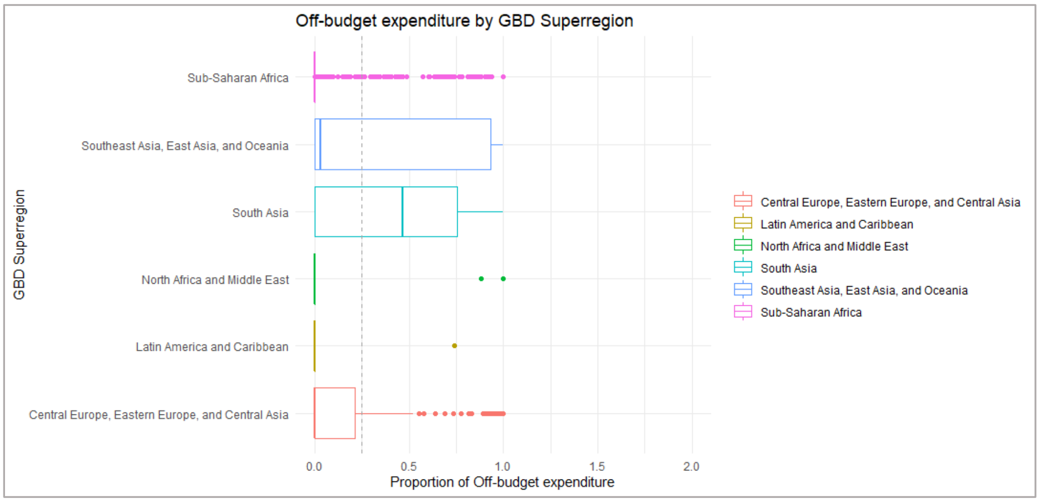

DAH_HSS: The proportion of DAH allocated to health systems strengthening (HSS) is summarized here by GBD super-region. Southeast Asia followed by North Africa accounted for the highest median proportion of HSS DAH.

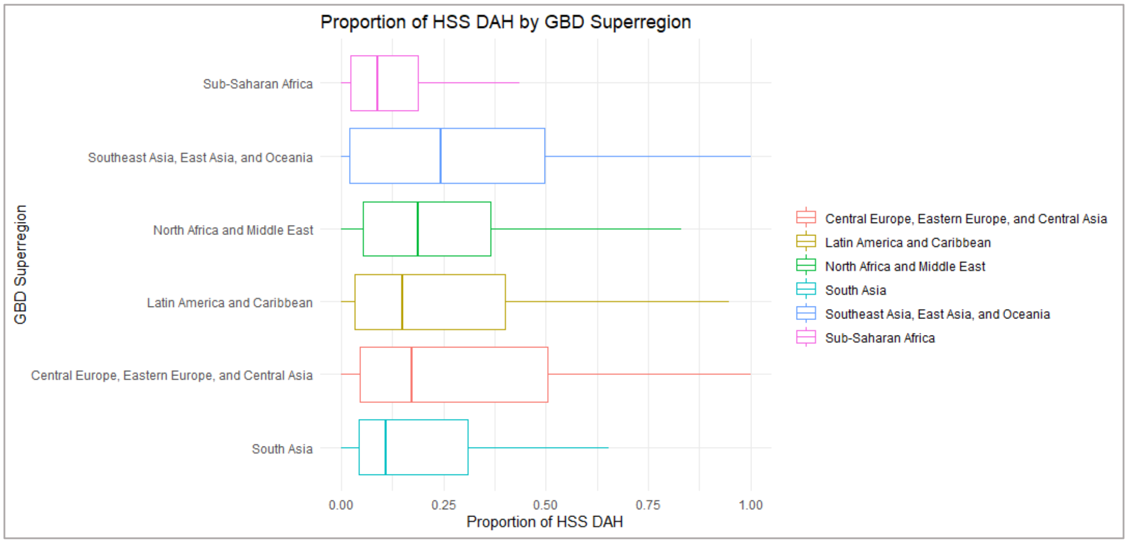

DAH from non-governmental sources: The proportion of DAH from non-governmental sources is summarized here by GBD super-region. Latin America and Sub-Saharan Africa accounted for the highest median proportion of DAH from non-governmental sources followed by the remaining GBD super-regions.

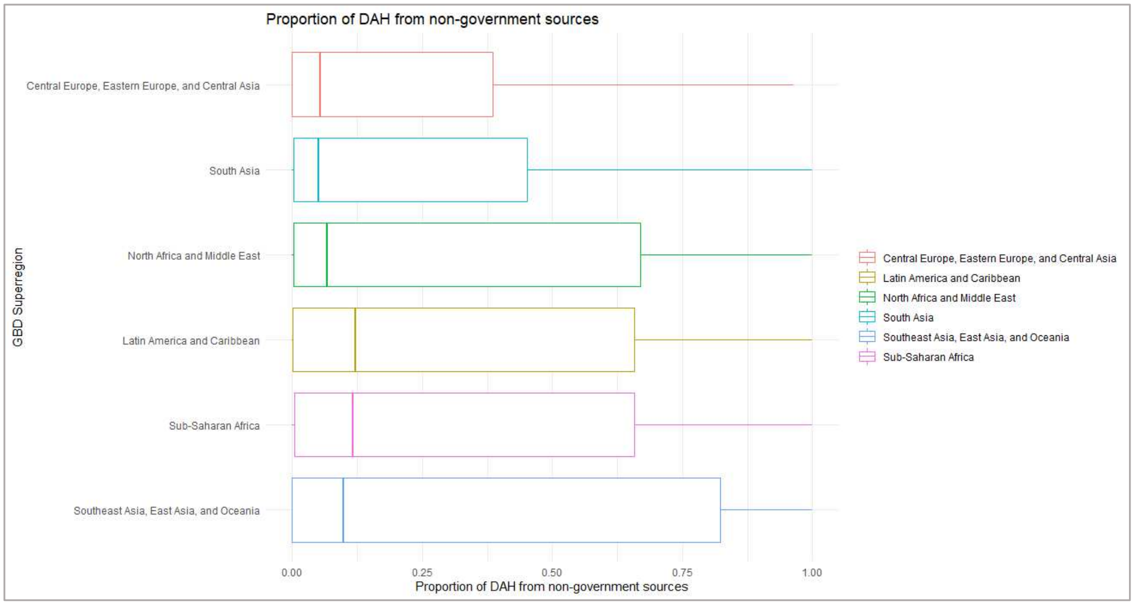

GDP: Gross domestic product per capita in US Dollars was estimated by IHME and summarized here by GBD super-region.

Moitra M, *et al. BMJ Global Health* 2021; 6:e004858. doi: 10.1136/bmjgh-2020-004858

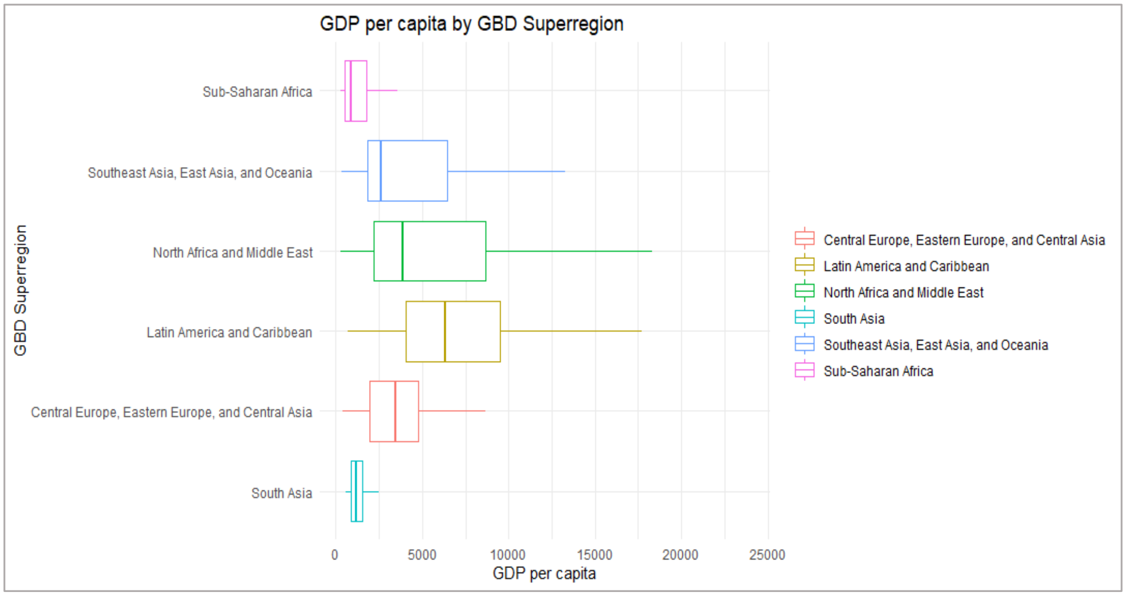

CRS Disbursement to Commitment: CRS ratios were calculated as CRS disbursement divided by CRS commitment for each country-year and summarized here by GDB super-region.

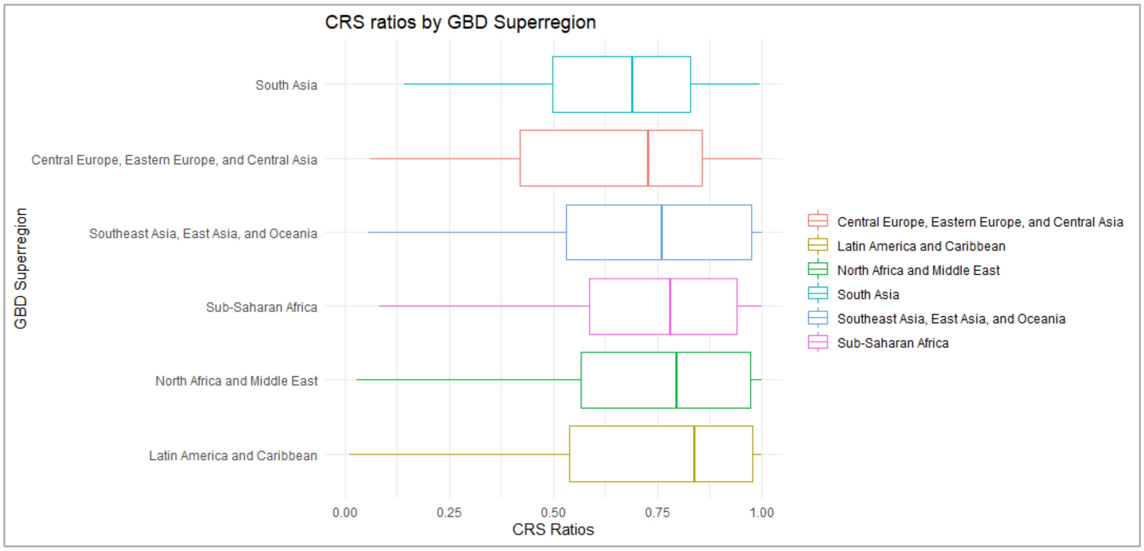

IHME DAH Disbursement to Commitment: IHME DAH Disbursement to Commitment were calculated and summarized here by super-region.

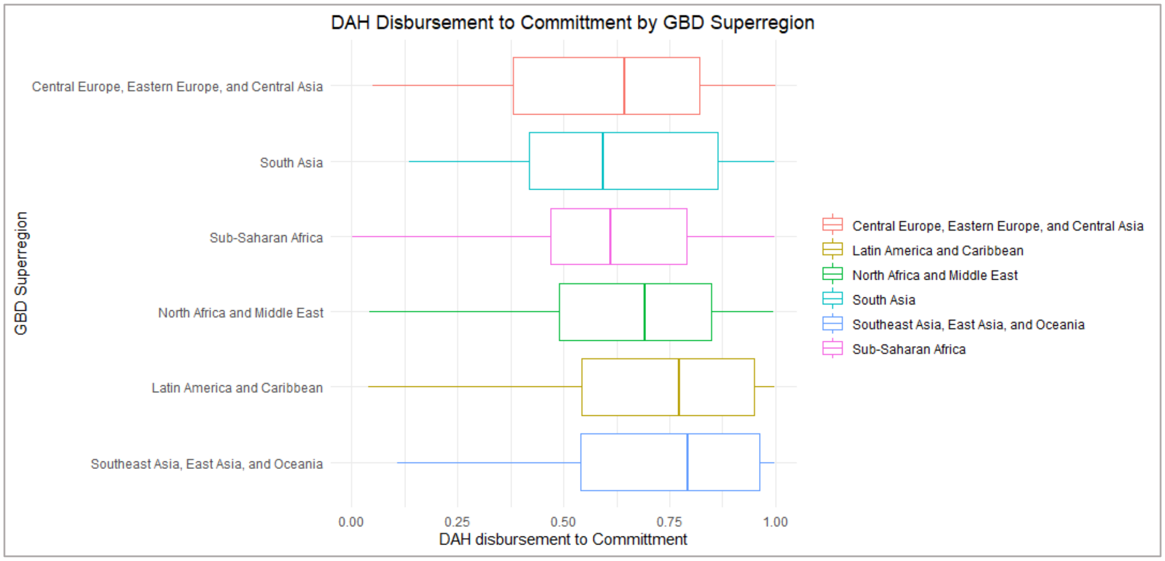

NHA Expenditure to CRS Commitment: NHA expenditure to CRS Commitment was calculated to model NHA data and are summarized here by GBD super-region.

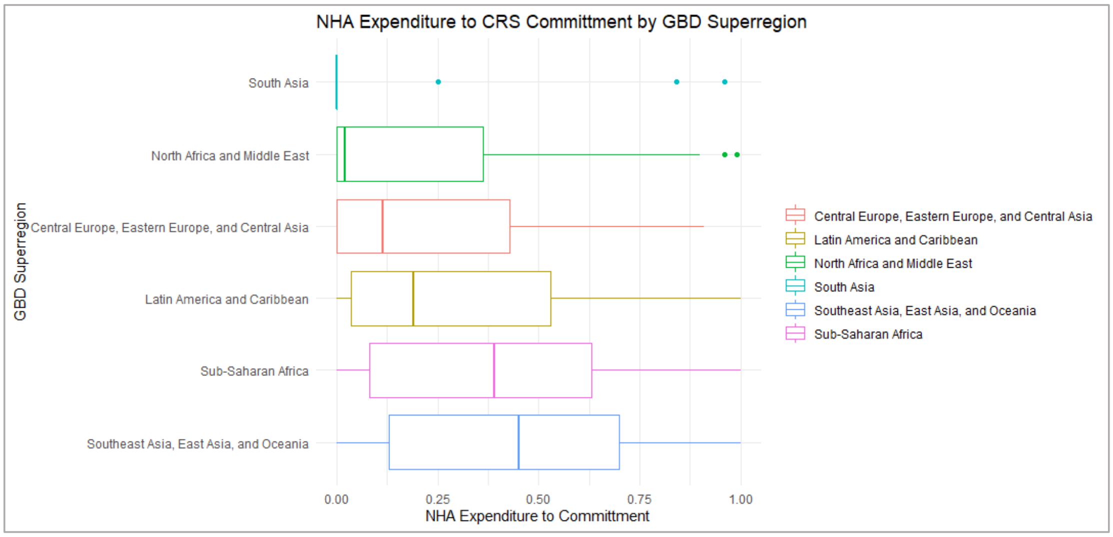

Additional results using FSI as a covariate

Parameter	CRS		IHME DAH		NHA	
	GBD super-region	Income-Level	GBD super-region	Income-Level	GBD super-region	Income-Level
Intercept	0.66 (0.03) ***	0.71 (0.02) ***	0.65 (0.05) ***	0.65 (0.05) ***	0.32 (0.04) ***	0.32 (0.02) ***
Off-budget > 25%	--	--	--	--	0.13 (0.05) *	0.21 (0.04) ***
In-kind > 15%	--	--	-0.072 (0.01) ***	-0.078 (0.014) ***	--	--
FSI	0.0004 (0.001)	0.0007 (0.0001)	-0.001 (0.0002) *	-0.001 (0.0002)	-0.0005 (0.001)	0.001 (0.001)
Proportion of HSS DAH	--	--	-0.03 (0.03)	-0.031 (0.03)	--	--
Proportion of NGO DAH	--	--	0.03 (0.02)	0.04 (0.01)	--	--
GDP	0.02 (0.01)	0.032 (0.02)	0.03 (0.008) ***	0.04 (0.01) **	-0.032 (0.02) *	-0.0003 (0.02)
Percent DAH from public sources	--	--	0.02 (0.04)	--	--	--
Latin America and the Caribbean	0.03 (0.03)	--	0.08 (0.03) **	--	0.03 (0.04)	--
North Africa and the Middle East	0.073 (0.04)	--	0.052 (0.03)	--	0.09 (0.05)	--
South Asia	0.007 (0.05)	--	0.04 (0.04)	--	0.25 (0.17)	--
Southeast Asia, East Asia, and Oceania	0.056 (0.03)	--	0.12 (0.03) ***	--	0.12 (0.05) *	--
Sub-Saharan Africa	0.08 (0.03) **	--	0.061 (0.02) *	--	0.12 (0.04) **	--
Low-Income	--	0.055 (0.04)	--	0.025 (0.03)	--	-0.13 (0.06) *
Low - Middle Income	--	-0.032 (0.02)	--	-0.03 (0.02)	--	0.14 (0.04) ***

Significance codes: 0 '***' 0.001 '**' 0.01 '*' 0.05
CRS (n = 1034); IHME (n = 1119); NHA (n = 1089)
GBD super-region reference level: Central Asia, Central Europe, Eastern Europe.
Income Group reference level: Upper Middle Income

Additional results using Income-level as a covariate

Parameter	CRS	IHME DAH	NHA
Intercept	0.72 (0.01) ***	0.64 (0.05) ***	0.32 (0.02) ***
Off-budget > 25%	--	--	0.18 (0.05) ***
In-kind > 15%	--	-0.07 (0.02) ***	--
CPI	0.002 (0.001) *	0.002 (0.001) ***	0.003 (0.001) *
Percent HSS DAH	--	-0.03 (0.03)	--
Percent NGO DAH	--	-0.03 (0.02)	--
GDP per capita	0.03 (0.02)	0.03 (0.01) *	-0.009 (0.02)
Percent DAH from public sources	--	0.084 (0.05)	--
Low-Income	-0.0016 (0.03)	0.03 (0.03)	0.171 (0.06) **
Low - Middle Income	0.03 (0.02)	-0.026 (0.02)	0.14 (0.04) ***
